# Plastics: From a Success Story to an Environmental Problem and a Global Challenge

**DOI:** 10.1002/gch2.202000026

**Published:** 2020-06-03

**Authors:** Rachid Dris, Seema Agarwal, Christian Laforsch

**Affiliations:** ^1^ LEESU UMR MA 102 UPEC École des Ponts Paris France; ^2^ Macromolecular Chemistry II University of Bayreuth Bayreuth Germany; ^3^ Animal Ecology I and BayCEER University of Bayreuth Bayreuth Germany

The 20th century witnessed the success of plastic materials. In just a few decades, these materials derived from petroleum chemistry became essential in our everyday life. A myriad of new products imposed themselves in countless daily applications and transformed our daily lifestyle profoundly. Several technical and medical innovations were made possible due to the versatile and adaptable material properties of plastics. To name only a few of these advantages, plastics are light yet stable, corrosion‐resistant, and have excellent insulating properties. Since the 1950s, the global production of plastics increased from 1.5 million tonnes to 359 million tonnes in 2018, growing by a factor of more than 200.

This success story came quickly with a significant downside, as plastic debris started to cause widespread pollution of the planet. In addition to the immediate anesthetic issues and the threat they represent for the biota, plastics can fragment into smaller particles, so‐called microplastics (<5mm), or even degrade further into nanoplastics. The occurrence of microplastics in marine systems was reported as early as 1972 and became a topic of interest for scientists in 2004. Since then, as exponentially as plastic production increased, concerns about this pollution grew. The scientific community plays a central role in tackling this global challenge, as it is an urgent matter to close the considerable gaps in knowledge that are becoming increasingly apparent. While the reports available to date are sufficient to establish the ubiquity of plastic debris, the questions appear more numerous than the answers as to the distribution, fate, and impacts of plastics in natural environments. This special issue features several papers presenting and discussing these global challenges.

The ubiquitous contamination of the environment by plastic waste and especially by microplastics, along with the potential risks it poses to ecosystems and to our health, have recently become the focus of public and scientific interest. Until a few years ago, the young research field of microplastics focused exclusively on the oceans. Since then, microplastics were encountered in freshwater, the air, soil, as well as in urban effluents (runoff, wastewater). However, a first challenge consists in the difficulty of providing reliable estimates of the levels of plastics input into the environment. While the role of wastewater treatment plant discharges is well known, the multiplicity of potential sources of microplastics and the lack of knowledge about the quantities released prevents the identification of the main contributors. Hence, further efforts are still required to estimate the relative contributions of the various sources and prioritize actions to reduce microplastic contamination. From the source to the environment, plastic debris does not follow a direct and linear path. A better understanding of the mechanisms governing the transport of this debris is necessary to estimate the residence time of plastics in different environments and settings. Moreover, plastics will undergo different processes like degradation, biofilm formation, and pollutant adsorption in the environment. Further work is required to better understand the fate of plastic pollution in all environmental compartments.

In this context, one of the major shortcomings met by the scientific community resides in the comparability of the studies. A heterogeneity is encountered from the plastics targeted (sizes and polymers considered) to the methods used. As this prevents coherent comparisons from being made, it limits the possibilities of learning lessons about the sources of plastics, their dynamics in the environment, their fate, and their impact. This lack of homogeneity does not stem from a lack of scientific effort, but rather from several aspects. As the microplastic research field is relatively young, methodological innovations and advancements are constantly improving. It is therefore counterproductive and too early to standardize a single, efficient method. Further, the various types of matrixes analyzed (marine water, freshwater, wastewater, sediments, air, soil, compost, etc.) require different approaches and adaptations of already existing methods. Finally, a big challenge exists in the difficulties met when trying to validate and evaluate the methods' efficiencies.

Plastic pollution was first encountered in the marine environment, which is still in the focus of most of the published studies to date. However, due to the lack of methodological validation and heterogeneity, the distribution of plastics in the marine environment is still poorly known. While attempts have been made to quantify the number and total mass of plastics present in the oceans, many questions remain about their validity. This special issue includes a review discussing the history and the impact of plastics in the marine environment, as well as the main future challenges related to this pollution.^[^ ^1^ ^]^

The ubiquitous contamination of the environment by microplastics may pose risks to ecosystems and to our health. However, the environmental impact is still poorly known. Ecotoxicological studies, for instance, often provide conflicting results on the potential impact of microplastics on organisms. This has led to an intense scientific debate since the beginning of the last decade. An improvement in practices and the production of comparable data appears vital in order to answer the crucial question of the impacts of plastic pollution not only on biota but also on human health. This challenge is discussed by one of the papers included in this special issue.^[^ ^2^ ^]^ This review highlights the challenges and limitations currently faced in the issue of microplastics in freshwater. Recommendations are provided for methodological standardization, particularly for microplastic characterization, quality assurance, and quality control (QA/QC) procedures as well as result reporting.

As microplastics further degrade, nanoplastics represent a largely unknown part of plastic pollution. These particles are too small to be detected with certainty by the currently available methods, and their quantity in the environment remains completely unknown. Moreover, it is suspected that their small size allows them to pass easily through biological barriers, thus inducing an increased ecotoxicological risk. Their detection and quantification, along with the study of their health impacts, are major research questions. A case study assessing the ingestion of nanoplastics by mussels and discussing these challenges is published within the framework of this special issue.^[^ ^3^ ^]^

In order to better highlight this global challenge and the uncertainties surrounding the microplastics issue, a debate between two scientists is also included, touching upon a broad range of issues like the differences in risk conceptions and communication, the scientific uncertainty and its consequences on risk assessment, the prioritizing of environmental issues, the costs of action and inaction, and the application of several approaches for policy‐making.^[^ ^4^ ^]^ Because of the uncertainty still surrounding the microplastic issue, as discussed here and in the papers published within this special issue, it is a great challenge for the scientific community to transfer knowledge to the public and respond to the public concerns about plastic pollution. Therefore, a study in this special issue examines how environmental risks related to microplastics are framed in peer‐reviewed publications on the one hand, and online newspaper articles dedicated to the general public on the other.^[^ ^5^ ^]^ It provides interesting perspectives into the risk perception of the public and decision makers.

Due to global population development, increasing urbanization, and rising consumption in developing and emerging countries, it is predicted that the production and consumption of plastics will continue to grow strongly on a global scale. The environmental issue of plastics is now a global challenge: for the scientific community, required to gain knowledge about the environmental fate of plastic waste and its ecological impact; for global media, needed to communicate about this issue to the general public; for public policies, essential for the development of operational responses; and for all citizens, who must adjust their consumption patterns and waste management. After all, plastics play an indispensable role in our everyday lives, for example in healthcare, and therefore it is not entirely the material itself that causes the global challange of plastic pollution, but how we deal with it.
